# Clinical outcomes after coronary artery bypass grafting in patients with dialysis-dependent end-stage renal disease and an analysis of the related influencing factors

**DOI:** 10.1007/s00380-023-02261-w

**Published:** 2023-03-21

**Authors:** Xi-hui Li, Si-yu Zhang, Feng Xiao

**Affiliations:** Department of Cardiovascular Surgery, Peking University First Hospital, Beijing, 100034 China

**Keywords:** End-stage renal disease, Dialysis-dependent, Coronary artery bypass grafting, Survival rate

## Abstract

Perioperative and short/mid-term survival rates of dialysis-dependent patients with end-stage renal disease (ESRD), who undergo coronary artery bypass grafting (CABG), and the factors influencing mortality are not well evaluated In China. We retrospectively analyzed the perioperative and postoperative 1-, 3-, and 5-year survival rates of 53 dialysis-dependent ESRD patients who underwent CABG, and compared the factors related to perioperative mortality and all-cause mortality during the postoperative follow-up. Survival rates were expressed as Kaplan–Meier survival curves, and factors influencing the follow-up survival rates were analyzed using the log rank (Mantel–Cox) test. There were eight perioperative deaths, resulting in 15.1% mortality. Intraoperative intra-aortic balloon pump use (*P* = 0.01), advanced age (*P* = 0.0027), and high EuroSCORE II score (*P* = 0.047) were associated with increased perioperative mortality. Forty-five discharged patients were followed from 2 months to 10 years (median, 4.2 years) postoperatively. There were 19 all-cause deaths, including 10 cardiac deaths (10/19, 52.6%). Comparisons between groups indicated that the presence of peripheral artery disease (PAD) increased mortality during follow-up (*P* = 0.025); 1-, 3-, and 5-year survival rates were 93.3, 79.5, and 66.8%, respectively. The results of the long-rank analysis indicated that the presence of PAD was a risk factor for postoperative survival (log rank χ^2^ = 4.543; *P* = 0.033). Dialysis-dependent patients with ESRD had high perioperative mortality and unsatisfactory short- and medium-term survival after CABG. PAD was a risk factor affecting patients’ postoperative survival. Multidisciplinary teamwork is needed to enhance postoperative management and reduce complications, to improve postoperative survival in these patients.

## Backgroud

**C**oronary artery bypass grafting (CABG) is an effective treatment for severe coronary atherosclerotic heart disease (coronary heart disease). Dialysis-dependent patients with end-stage renal disease (ESRD) and pathophysiological changes secondary to severe renal insufficiency (such as diffuse coronary atherosclerosis, aortic calcification, calcium and phosphorus metabolism disorders, lipid metabolism disorders, anemia, platelet dysfunction, and impaired water and electrolyte balance) are a high-risk population for cardiac surgery. Perioperative mortality in this patient population is significantly higher than that of patients with normal renal function, and cardiac surgery mortality is 3.9 times higher than for those without ESRD [1]. Factors related to the high mortality are controversial and have no confirmatory data in China. Therefore, we retrospectively analyzed the perioperative and follow-up all-cause mortality of 53 patients with dialysis-dependent ESRD who underwent consecutive CABG in the Department of Cardiac Surgery, Peking University First Hospital from September 2004 to September 2018, as well as the factors influencing mortality.

## Methods

### Study population and design

We enrolled dialysis-dependent ESRD patients undergoing CABG who were admitted to the Department of Cardiac Surgery of Peking University First Hospital from September 2004 to September 2018. Data were collected from inpatient and routine outpatient follow-up records. Inclusion criteria were: 1) dialysis-dependent patients with ESRD diagnosed preoperatively; and 2) patients undergoing consecutive CABG for severe coronary artery disease that could be associated with valvular disease requiring concurrent surgery. Exclusion criteria were: 1) incomplete clinical data; and 2) loss of follow-up in the outpatient clinic, postoperatively.

We collected the following preoperative clinical data: sex, age, diabetes, hypertension, hyperlipidemia, peripheral artery disease (PAD), history of percutaneous coronary intervention (PCI), chronic obstructive pulmonary disease, body mass index, left ventricular ejection fraction (LVEF), cardiac function classification (New York Heart Association cardiac function classification), dialysis type (hemodialysis/peritoneal dialysis), dialysis-dependent time, preoperative serum creatinine, EuroSCORE II score, emergency surgery, cardiopulmonary bypass, preoperative or intraoperative intra-aortic balloon pump (IABP) use, delayed extubation (mechanical ventilation > 24 h), use of the internal mammary artery, number of graft vessel bridges, and prolonged intensive care unit (ICU) stay (> 72 h). Using all-cause death as the endpoint, we analyzed perioperative and 1-, 3-, and 5-year follow-up data to calculate patients’ perioperative and postoperative survival rates and to analyze the related influencing factors.

### Statistical analyses

All measurement data are expressed as mean ± standard deviation. Measurement data between the two groups were analyzed using the t-test or nonparametric rank sum test, and numerical data were analyzed using the *χ*^2^ test or Fisher's exact test. Survival rates were expressed using Kaplan–Meier survival curves, and factors influencing survival were analyzed using the long-rank (Mantel–Cox) test. Statistical analysis was performed using SPSS 20.0 software (IBM Corp., Armonk, NY). *P* < 0.05 indicated a significant difference.

## Results

### Baseline and surgical data

A total of 53 dialysis-dependent ESRD patients who underwent CABG were included in this study. Causes of renal failure were as follows: diabetic nephropathy (*n* = 22), hypertensive nephropathy (*n* = 20), aristolochic acid nephropathy (*n* = 2), and IgA nephropathy, glomerulonephritis, renal artery stenosis, renal vasculitis, and other causes (*n* = 9). There were 41 men and 12 women aged 46–84 years (62.6 ± 9.1 years), constituting 48 patients with hypertension, 24 with diabetes, and 24 with peripheral vascular disease. Thirty-eight patients received hemodialysis and 15 received peritoneal dialysis. Patients were dialysis-dependent for a minimum of 1 week and a maximum of 13 years (3.5 ± 3.8 years). Acute myocardial infarction was diagnosed in 18 patients, unstable angina in 35 patients, and coronary angiography showed left main coronary artery lesions or three-vessel lesions or both. Six patients had valvular disease requiring concurrent surgery (four for severe mitral regurgitation, one for severe aortic stenosis, the last one for severe mitral valve and moderate aortic valve regurgitation). All the patients had no thoracic aorta aneurysm. Preoperative serum creatinine level ranged from 361 μmol/L to 1135 μmol/L (average, 712 ± 234 μmol/L).

Four patients underwent emergency CABG because of acute myocardial infarction, and 49 patients underwent elective surgery. All patients underwent conventional tracheal inhalational and intravenous anesthesia, and thoracotomy was performed at the mid-sternum. Forty-four patients underwent off-pump CABG, and 9 patients underwent off-pump CABG and/or valve replacement.

Preoperative hemodialysis patients underwent routine hemodialysis three times a week, and peritoneal dialysis patients underwent dialysis daily. All patients underwent hemodialysis 1 day before surgery, and continuous hemofiltration was performed immediately without surgical bleeding, 2–3 h postoperatively. After the patient was stable and transferred from the ICU, routine preoperative hemodialysis or peritoneal dialysis was resumed.

### Perioperative results

Eight patients died during perioperatively, leading to a mortality rate of 15.1%. The causes of death are shown in Table 1.

**Table 1:** Perioperative causes of death

Cause of death	Number of deaths (*n*)
Infection toxic shock	2
Massive cerebral infarction	2
Multiple organ failure	2
Aspiration	1
Superior mesenteric artery embolism	1

Eight patients died and were assigned to the perioperative death group, and the 45 patients who survived were assigned to the perioperative survival group. Comparisons of the clinical data between the two groups are shown in Table 2. The results indicated that IABP (*P* = 0.01), advanced age (*P* = 0.0027), and high EuroSCORE II score (*P* = 0.047) were associated with increased perioperative mortality.

**Table 2:** Comparisons of the clinical data between the perioperative death group and the survival

Clinical information	Death group (8)	Survival group (45)	*t*/χ^2^	*P*
Male/Female	8/0	33/12	–	0.175
Age (years)	69.1 ± 11.3	61.5 ± 8.3	2.273	0.027†
Dialysis-dependent time (years)	5.4 ± 4.1	3.2 ± 3.6	1.441	0.183
Hypertension	7	41	–	0.574
Diabetes	5	19	–	0.444
Hyperlipemia	3	15	–	0.521
COPD	1	19	–	0.234
PCI	1	6	–	1.000
Cardiac function	3 ± 1.2	2.6 ± 0.8	1.358	0.180
LVEF (%)	49.6 ± 18.9	55.3 ± 15.4	0.923	0.360
BMI	23.7 ± 2.05	23.4 ± 3.1	0.285	0.777
HD/PD	4/4	34/11	–	0.202
LVEF ≤ 40%	4	11	–	0.202
PAD	3	22	–	0.715
Preoperative Scr (μmol/L)	702 ± 240	739 ± 220	0.241	0.822
EUROSCOREII	14.3 ± 11.3	4.3 ± 3.5	2.397	0.047†
Emergency	2	2	–	0.104
Combined valvular surgery	1	5	–	1.000
Extracorporeal circulation	1	8	–	1.000
IABP	4	1	–	0.001†
Internal mammary artery	4	30	0.810	0.450
Number of graft bridges	2.4 ± 0.8	2.6 ± 0.7	0.620	0.532

^†^Significant difference; *COPD* chronic obstructive pulmonary disease, *PCI* percutaneous coronary intervention, *LVEF* left ventricular ejection fraction, *BMI* body mass index, *HD* hemodialysis, *PD* peritoneal dialysis, *PAD* peripheral artery disease, *Scr* serum creatinine level, *IABP* intra-aortic balloon pump

### Postoperative follow-up results

We followed the 45 discharged patients for a median of 4.2 years, with a minimum of 2 months (died 2 months postoperatively) and a maximum of 10 years. There were 19 all-cause deaths, including 10 cardiac deaths (10/19, 52.6%). Three patients died of digestive tract diseases (one gastrointestinal bleeding, one severe cirrhosis, one intestinal obstruction), and three died of infectious diseases (one pneumonia, one diabetic foot gangrenous infection, and one abdominal infection after kidney transplantation). The causes of death are shown in Table 3.

**Table 3:** The causes of death during follow-up

Cause of death	Number of death (*n*)
Acute myocardial infarction	7
Cardiac failure	3
Cerebral hemorrhage	1
Malignant tumor	1
Disease of digestive tract	3
Hyperkalemia	1
Infectious diseases	3

We classified patients surviving during the follow-up as the follow-up survival group, and those who died as the follow-up death group. Table 4 shows the data comparisons between the two groups. The results of the comparisons indicated that the presence of peripheral vascular disease was associated with increased postoperative mortality rates (*P* = 0.025).

**Table 4:** Comparisons of clinical data between the follow-up death group and the follow-up survival group

Clinical information	Follow-up death (19)	Follow-up survival (26)	*t*/χ^2^	*P*
Male/Female	13/6	20/6	0.406	0.524
Age (years)	60.8 ± 10	62.0 ± 7.1	0.480	0.634
Dialysis-dependent time (years)	3.0 ± 3.8	3.2 ± 3.6	0.163	0.872
Hypertension	17	24	0.109	0.741
Diabetes	8	11	0.000	0.989
Hyperlipemia	5	10	–	0.589
COPD	8	11	–	0.989
PCI	1	5	–	0.222
PAD	9	13	5.021	0.025†
Cardiac function	2.7 ± 0.7	2.4 ± 0.9	1.338	0.789
LVEF (%)	54.5 ± 17.7	55.9 ± 14.0	0.286	0.776
BMI	23.2 ± 3.1	23.2 ± 3.1	0.222	0.983
HD/PD	14/5	20/6	–	1.000
LVEF ≤ 40%	5	6	–	0.720
Preoperative Scr (μmol/L)	702 ± 240	739 ± 220	0.241	0.822
EUROSCOREII	4.7 ± 4.4	4.0 ± 2.7	0.599	0.554
Emergency	2	0	–	0.173
Combined valvular surgery	3	2	–	1.000
Extracorporeal circulation	1	8	–	1.000
IABP	1	0	–	0.422
Delayed extubation (> 24 h)	5	5	–	0.720
ICU duration (> 72 h)	14	14	1.838	0.175
Internal mammary artery	13	17	0.920	0.812
Number of graft bridges	2.5 ± 0.9	2.6 ± 0.6	0.720	0.501

^†^Significant difference, *COPD* chronic obstructive pulmonary disease, *PCI* percutaneous coronary intervention, *LVEF* left ventricular ejection fraction, *BMI* body mass index, *HD* hemodialysis, *PD* peritoneal dialysis, *PAD* peripheral artery disease, *IABP* intra-aortic balloon pump

**Fig. 1:**
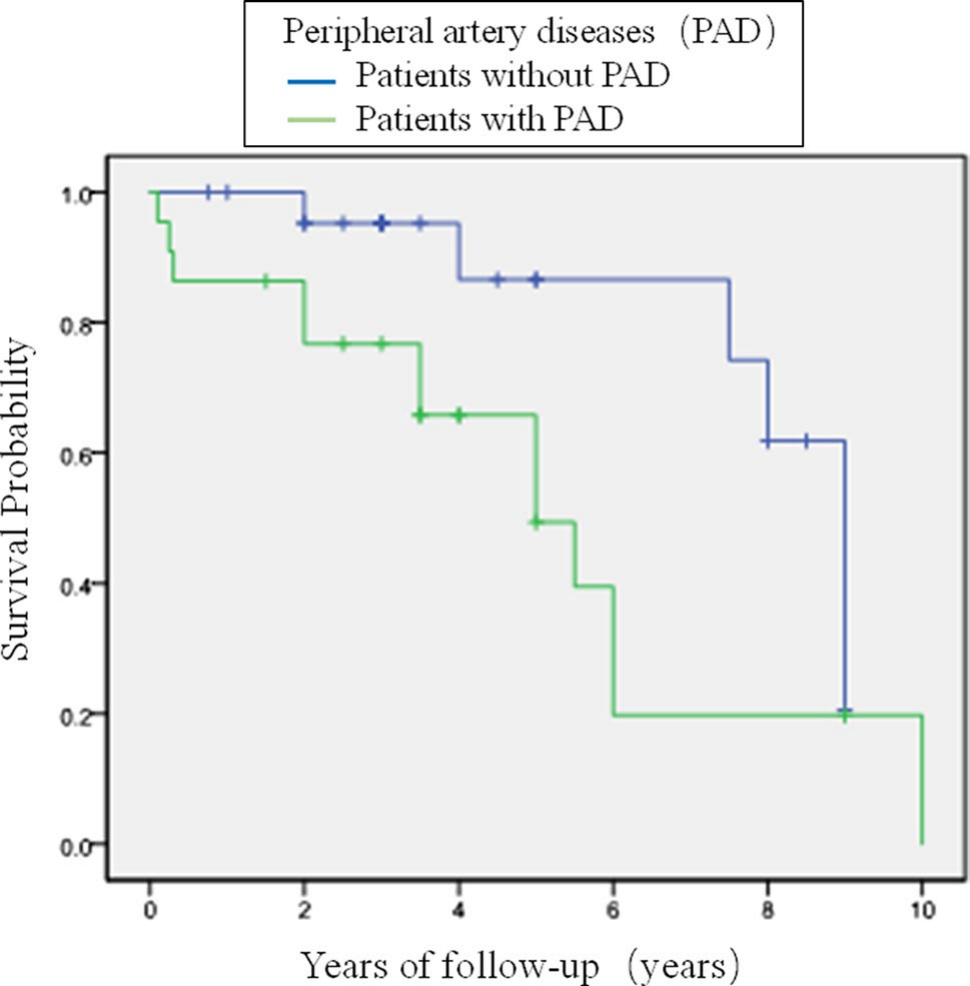
Survival rates estimated by Kaplan–Meier analysis

We used Kaplan–Meier survival curves to calculate the survival rate of the discharged patients; 1-, 3-, and 5-year survival rates were 93.3%, 79.5%, and 66.8%, respectively. We used the clinical data in Table 4 in the Kaplan–Meier survival analysis, and we used the log rank test to analyze the factors influencing postoperative survival. Results showed that PAD was the only risk factor for postoperative survival (log rank χ^2^ = 4.543, *P* = 0.033). The survival curve is shown in Fig. 1.

## Discussion

With improved dialysis techniques, the number of patients surviving after developing ESRD has increased significantly, and nearly 50% of the patients eventually die of cardiovascular diseases, rather than renal disease.

### CABG or PCI

CABG is an effective treatment for severe coronary heart disease. Although to our knowledge, there are no randomized controlled studies evaluating dialysis-dependent patients with ESRD and coronary heart disease, a retrospective analysis involving 1015 dialysis-dependent patients with ESRD showed that CABG was significantly superior to PCI regarding postoperative revascularization, and long-term all-cause mortality was at least equivalent to PCI [2]. At the 5-year follow-up, CABG was associated with significantly lower rates of cardiac death, sudden death, myocardial infarction, and revascularization compared with PCI, although there was no significant difference for all-cause deaths [3]. The perioperative risk of CABG is higher than that of PCI, but patients benefit regarding long-term survival [4]. Therefore, for patients with ESRD with left main or/and three-vessel coronary lesions who can undergo both PCI and CABG, we prefer CABG.

### Influence factors of peri-operative death

In this study, eight patients died perioperatively, leading to a mortality rate of 15.1%. Regarding the factors associated with mortality, we found that IABP use (*P* = 0.01), advanced age (*P* = 0.0027), and high EuroSCORE II score (*P* = 0.047) were associated with higher perioperative mortality. Studies have shown that ESRD with dialysis dependence is a predictor of in-hospital mortality (odds ratio: 3.1, *P* < 0.001), and significantly increased perioperative cardiac mortality, postoperative sepsis, and respiratory failure, [1] similar to the results in our study. Multiple factors are related to perioperative mortality, namely, surgical complexity, preoperative cardiac function grade IV (New York Heart Association classification), preoperative acute myocardial infarction, combined surgery, aortic surgery, age ≥ 70 years, history of heart failure, PAD, female sex, dialysis dependence ≥ 5 years, emergency surgery, and preoperative hypertension [1, 5–7]. Jault et al. retrospectively analyzed the results of cardiac surgery in 124 patients receiving dialysis. Of these patients, 46% constituted the coronary heart disease group (group 1), 29.8% constituted the valvular disease group (group 2), 14.5% constituted the coronary heart disease and valvular disease group (group 3), and 9.6% constituted the high-risk disease group (group 4) with conditions such as emergency, secondary, and complex diseases; aortic dissection; aortic aneurysm; and prosthetic valve endocarditis; 14.5% of patients had diabetes mellitus, resulting in an overall mortality of 16.9%. Age, sex, hypertension, diabetes mellitus, preoperative myocardial infarction, disease type, preoperative ejection fraction, and 30-day postoperative mortality were analyzed in Jault et al.’s study. The only risk factor was complex cardiac surgery. The combined overall mortality rate for groups 3 and 4 was significantly higher than the combined mortality rate for groups 1 and 2 (30 vs. 12.7%, respectively) [5].

### Influence factors of follow-up death

During the follow-up of our 45 discharged patients, the shortest follow-up period was 2 months (died 2 months postoperatively), and the longest was 10 years, with a median of 4.2 years. There were 19 all-cause deaths, including 10 cardiac deaths (10/19, 52.6%). The 1-, 3-, and 5-year survival rates were 93.3, 79.5, and 66.8%, respectively. Log rank analysis of the factors influencing patients' postoperative survival showed that PAD was the only risk factor affecting survival. Our results also indicated that even after CABG surgery, more than 50% of dialysis-dependent ESRD patients eventually died of cardiovascular diseases (acute myocardial infarction and heart failure), and that PAD was a risk factor for long-term survival. The overall survival rates were 76.9%, 60.0%, 43.9%, and 36.2% at 3, 5, 8, and 10 years, respectively. In the Kaplan–Meier model, multivariate analysis showed that age ≥ 63 years (*p* = 0.014), diabetes mellitus (*p* = 0.036), and PAD (*p* = 0.044) were predictors of late death, and that diabetes mellitus (*p* = 0.038) and LVEF ≤ 0.40 (p = 0.027) were predictors of late cardiovascular events. Thus, intensive symptomatic and supportive treatment may be needed for patients older than 63 years with diabetes, PAD, and low LVEF [8]. Sezai et al. reported that the postoperative survival rates were 81.5 ± 18.5% at 1 year, 72.0 ± 28.0% at 5 years, and 68.4 ± 31.6% at 8 years. Logistic regression analysis showed that acute myocardial infarction, age ≥ 75 years, preoperative IABP implantation, and combined surgery were risk factors for early death. In particular, the prognosis of patients with preoperative left ventricular dysfunction, and IABP-assisted and combined surgery was poor [9]. In a retrospective analysis of 483 consecutive cardiac operations, patients with valvular disease, active endocarditis, and low left ventricular function had increased mid-term mortality [10]. Therefore, for such patients, multiple related factors affect postoperative survival, and cardiovascular disease is still the main cause of death. To improve the surgical effect, improving CABG surgical technique and postoperative comprehensive treatment by a multidisciplinary team are needed to decrease complications in the cardiovascular system and other systems.

### Choice of surgical technique

Because ESRD patients experience many complications after cardiopulmonary bypass, we prefer to use an off-pump technique and graft using the internal mammary artery, as much as possible. The rates of perioperative complications following off-pump CABG, such as bleeding, allogeneic blood transfusion, duration of mechanical ventilation, positive inotropic drug support, perioperative myocardial infarction, and new atrial fibrillation, are lower than those for CABG under traditional cardiopulmonary bypass [11]. Shroff et al. reported of the 13 085 dialysis patients (2001–2006) in the US Renal Data System database, 2335 (17.8%) patients underwent off-pump CABG. Off-pump CABG significantly reduced all-cause mortality (hazard ratio 0.92, 95% CI 0.86–0.99; *P* = 0.02), with the most obvious benefit occurring in the first year postoperatively. There was no difference in all-cause mortality between the two groups in the second year follow-up. Additionally, no difference was found between the groups for cardiovascular mortality, and the use of the internal mammary artery significantly improved postoperative survival (hazard ratio 0.92; 95% CI 0.87–0.98; *P* = 0.0057) [12]. Compared with CABG under conventional extracorporeal circulation, CABG without cardiopulmonary bypass can achieve better long-term survival [13]. Some studies suggested that off-pump CABG has no significant advantage for dialysis-dependent patients. Ten retrospective studies (2762 patients undergoing off-pump CABG, and 11 310 patients undergoing CABG under traditional cardiopulmonary bypass) reported no significant difference in early mortality, hemostasis by thoracotomy, allogeneic blood transfusion rates, stroke, or atrial fibrillation between the two surgical methods. Patients undergoing off-pump CABG were extubated earlier (*p* < 0.01). There was also no difference in 3-year survival postoperatively [14], and no difference in mortality, myocardial infarction, stroke, or revascularization rates, even at 30 days, 1 year, and throughout the follow-up period [15]. Most studies confirmed the use of the internal mammary artery (17, 18). For patients with multivessel disease and an average follow-up of 2.5 years, bilateral internal mammary arteries skeletonized in situ decreased all-cause mortality (*p* = 0.02) and cardiovascular mortality (*p* = 0.04) [16]. At 3-, 5-, 7-, and 10 years of follow-up (mean, 5.2 years) in 130 ESRD patients undergoing CABG, the use of bilateral internal mammary arteries significantly reduced the rate of cardiovascular events in ESRD patients without diabetes (*p* = 0.0143) [17].

But for the patients with valvular disease requiring concurrent surgery, cardiopulmonary bypass became necessary. The indications of concurrent operation included: obvious clinical symptoms, moderate or severe regurgitation or/and stenosis, left ventricular enlargement or LVEF decreased (LVEF≦50%). More transfusion were needed for concurrent surgery than isolate CABG. The 6 patients with valvular disease had no surgical contraindications, so the transcatheter aortic valve implantation (TAVI) procedure wasn’t considered.

### Peritoneal dialysis or hemodialysis

The results of our study showed that peritoneal dialysis and hemodialysis had no effect on perioperative and short- or medium-term prognosis, which was consistent with the survival results of Kumar et al. [19]. There was no difference in preoperative general data between the 36 patients receiving peritoneal dialysis and the 72 patients receiving hemodialysis, or in 2-year survival rate postoperatively, but the number of patients experiencing perioperative complications such as postoperative infection and delayed extubation, and the number who died were higher in the hemodialysis group [18]. It has also been reported that both in-hospital and 1-year postoperative mortality rates in peritoneal dialysis patients are higher than for hemodialysis patients. In one study, among the patients who died in-hospital, more hemodialysis patients died from cardiac events vs patients receiving peritoneal dialysis who died from septic toxic shock [19].

## Conclusions

Patients with dialysis-dependent ESRD who underwent CABG had high perioperative mortality (15.1%) and unsatisfactory short- and medium-term survival rates (5-year survival rate, 66.8%), in our study. Cardiovascular causes were the main reason for all-cause mortality (52.6%), and preoperative PAD was associated with increased postoperative mortality. Multidisciplinary teamwork is needed to enhance postoperative management, reduce complications, and improve postoperative survival rates in dialysis-dependent ESRD patients after CABG.

This study has potential limitations. The effect estimates are based on a small-sample retrospective study. There are, therefore, biases and confusions that may have influenced our effect estimates. The first is there are only 53 cases in our study. The operations with a large number of cases of dialysis-dependent ESRD have not been performed in China, because both the cardiac surgeons and patients are afraid of the high mortality and morbidities. The second is our study is a retrospective study. So the two limitations may influence the results. It is a good start and we tried our best to improve the operative effect, however, there is still a long way to go. We plan to strengthen publicity campaign on the procedures, thus more surgeons and patients would accept it. In our next prospective study, more cases and other heart centers will be included. We believe that a perfect effect will help surgeons and patients.

## Data Availability

Please contact author for data requests.
